# Supplementary material for: Tumor-Infiltrating CD20+ B Lymphocytes: Significance and Prognostic Implications in Oral Cancer Microenvironment
Source: Cancers (Basel). 2021 Jan 21;13(3):395. doi: 10.3390/cancers13030395 (PMC7865920; doi:10.3390/cancers13030395)
Supplement: Supplementary file 1 [file cancers-13-00395-s001.pdf]

Tumor-Infiltrating CD20+ B Lymphocytes: Significance and Prognostic Implications in Oral Cancer Microenvironment

Faustino Julián Suárez-Sánchez, Paloma Lequerica-Fernández, Juan Pablo Rodrigo, Francisco Hermida-Prado, Julián Suárez-Canto, Tania Rodríguez-Santamarta, Francisco Domínguez-Iglesias, Juana M. García-Pedrero and Juan Carlos de Vicente

Table S1. Summary of immune infiltration levels (mean numbers) in the tumor and surrounding stroma of 125 OSCC patients.

Tumor-infiltrating Immune Subtypes (cells per mm^2)	Tumor Nests Mean ± SD (range)	Stroma Mean ± SD (range)
**CD4$^+$ TILs**	6.02 ± 12.29 (0.00 to 86.33)	54.60 ± 68.15 (1.33 to 569.67)
**CD8$^+$ TILs**	47.88 ± 57.16 (0.00 to 288.33)	178.45 ± 203.21 (0.33 to 1202.67)
**FOXP3$^+$ Tregs**	3.11 ± 6.47 (0.00 to 50.00)	15.65 ± 24.54 (0.00 to 147.33)
**CD68$^+$ macrophages**	51.11 ± 45.08 (0.00 to 194.00)	122.72 ± 82.49 (9.67 to 488.67)
**CD163$^+$ macrophages**	31.11 ± 28.91 (0.00 to 187.33)	168.14 ± 97.59 (14.33 to 476.67)

Table S2. Associations between stromal and tumoral CD20/CD68 and CD20/CD168 ratios and clinicopathological parameters in the cohort of 125 OSCC patients.

Variable Mean (SD)	Number	Stromal CD20/CD68 Ratio	*p*	Tumoral CD20/CD68 Ratio	*p*	Stromal CD20/CD163 Ratio	*p*	Tumoral CD20/CD163 Ratio	*p*
Age (years)									
<65	77	0.29 (0.50)	0.31	0.04 (0.12)	0.24	0.19 (0.39)	0.29	1.10 (3.97)	0.92
≥65	48	0.53 (1.14)		0.10 (0.36)		0.36 (0.79)		0.73 (2.25)	
Gender									
Female	43	0.52 (1.22)	0.71	0.11 (0.38)	0.28	0.33 (0.83)	0.96	0.18 (0.62)	0.25
Male	82	0.30 (0.48)		0.03 (0.11)		0.22 (0.39)		0.07 (0.19)	
Tobacco									
No	41	0.47 (1.14)	0.47	0.05 (0.16)	0.12	0.34 (0.83)	0.36	0.09 (1.73)	0.11
Yes	84	0.34 (0.59)		0.06 (0.27)		0.21 (0.40)		0.11 (0.47)	
Alcohol									
No	56	0.41 (1.05)	0.52	0.08 (0.33)	0.16	0.28 (0.74)	0.77	0.08 (0.16)	0.25
Yes	69	0.36 (0.55)		0.03 (1.28)		0.23 (0.42)		0.13 (0.53)	
pT									
T1 + T2	81	0.47 (0.95)	0.03	0.07 (0.28)	0.05	0.32 (0.69)	0.03	0.13 (0.46)	0.14
T3 + T4	44	0.21 (0.41)		0.03 (0.15)		0.14 (0.28)		0.07 (0.24)	
pN									
N0	76	0.43 (0.99)	0.51	0.07 (0.29)	0.87	0.30 (0.71)	0.76	0.12 (0.48)	0.94
N+	49	0.30 (0.41)		0.04 (0.14)		0.18 (0.25)		0.08 (0.23)	
Stage									
I + II	52	0.54 (1.14)	0.48	0.10 (0.35)	0.12	0.39 (0.84)	0.30	0.16 (0.57)	0.23
III + IV	73	0.26 (0.42)		0.03 (0.12)		0.16 (0.26)		0.07 (0.20)	
Grade									
Well	80	0.48 (0.98)	0.02	0.07 (0.28)	0.52	0.34 (0.70)	0.03	0.12 (0.46)	0.24
Moderate + Poor	45	0.21 (0.31)		0.04 (0.15)		1.19 (1.17)		0.08 (0.24)	
Site									
Tongue	51	0.36 (0.59)	0.61	0.07 (0.32)	0.67	0.26 (0.46)	0.74	0.05 (0.10)	0.75
Other	74	0.39 (0.94)		0.05 (0.17)		0.25 (0.65)		0.14 (0.51)	
Recurrence									
No	71	0.31 (0.53)	0.30	0.03 (0.12)	0.95	0.18 (0.31)	0.29	0.12 (0.51)	0.84
Yes	54	0.47 (1.08)		0.09 (0.34)		0.35 (0.80)		0.09 (0.17)	
Second primary tumor									
No	106	0.32 (0.80)	0.01	0.04 (0.14)	0.25	0.24 (0.61)	0.01	0.10 (0.43)	0.17
Yes	19	0.71 (0.84)		0.15 (0.52)		0.36 (0.37)		0.11 (0.16)	

All *p*-values were calculated using the U Mann-Whitney test.

Table S3. Univariate Cox regression analysis of disease-specific survival (DSS) in 125 patients with OSCC.

Variable	Number	Censored Patients (%)	DSS (95% CI)	*p*	Hazard Ratio	95% CI
Age						
<65 years	77	46 (60)	142.32 (118.63–166.01)	0.23	1.39	0.80–2.42
≥65 years	48	26 (54)	91.11 (70.43–111.79)			
Gender						
Female	43	25 (58)	130.56 (99.15–161.97)	0.77	1.08	0.61–1.92
Male	82	47 (57)	131.61 (107.28–155.94)			
Tobacco						
No	41	24 (59)	106.57 (83.24–129.91)	0.97	0.98	0.55–1.76
Yes	84	48 (57)	132.42 (108.70–156.14)			
Alcohol						
No	56	31 (55)	125.45 (98.13–152.76)	0.53	0.84	0.49–1.44
Yes	69	41 (59)	136.11 (109.92–162.29)			
pT						
T1–T2	81	53 (65)	151.82 (129.03–174.99)	0.001	2.49	1.44 – 4.30
T3–T4	44	19 (43)	77.62 (54.19–101.04)			
pN						
N0	76	49 (65)	127.96 (109.43–146.49)	0.01	1.92	1.12–3.31
N+	49	23 (4)	108.58 (77.88–139.28)			
Stage						
I + II	52	36 (69)	140.09 (120.15–160.04)	0.002	2.40	1.33–4.32
III + IV	73	36 (49)	113.33 (87.73–138.93)			
Grade						
Well	80	44 (55)	127.85 (103.73–151.98)	0.59	0.85	0.48–1.52
Moderate + Poor	45	28 (62)	121.63 (96.25–147.01)			
Site						
Tongue	51	28 (55)	124.43 (94.00–154.86)	0.31	0.75	0.43–1.30
Other	74	44 (60)	101.48 (123.52–139.47)			
Radiotherapy						
No	50	44 (88)	167.58 (150.15–185.00)	<0.0001	6.58	2.81–15.41
Yes	75	28 (37)	95.89 (73.23–118.55)			
Stromal $CD20^+$ TILs						
≤12.33	61	36 (59)	131.31 (104.39–158.23)	0.95	0.98	0.57–1.68
>12.33	64	36 (56)	132.74 (106.95–159.00)			
Tumoral $CD20^+$ TILs						
≤0.33	57	28 (49)	99.07 (76.37–121.78)	0.04	0.58	0.34–1.00
>0.33	68	44 (65)	148.94 (123.52–174.35)			
Stromal $CD20^+$/$CD8^+$ ratio						
≤0.1003	63	37 (59)	115.86 (94.45–137.28)	0.94	1.02	0.59–1.74
>0.1003	62	35 (57)	134.60 (108.12–161.07)			
Tumoral $CD20^+$/$CD8^+$ ratio						
≤0.0096	59	31 (53)	105.31 (83.00–127.63)	0.23	0.71	0.40–1.24
>0.0096	59	37 (63)	142.67 (114.81–170.54)			
Stromal $CD20^+$/$CD4^+$ ratio						
≤0.3136	65	36 (55)	126.46 (99.20–153.73)	0.33	0.76	0.44–1.32
>0.3136	60	36 (60)	118.54 (98.73–138.35)			
Tumoral $CD20^+$/$CD4^+$ ratio						
≤0.0909	47	26 (55)	126.83 (94.91–158.76)	0.36	0.75	0.40–1.40
>0.0909	48	29 (60)	134.30 (105.21–163.39)			
Stromal $CD20^+$/$FOXP3^+$ ratio						
≤0.7975	49	33 (67)	156.99 (127.91–186.07)	0.14	1.54	0.85–2.78
>0.7975	76	39 (51)	103.26 (85.13–121.39)			

Tumoral CD20+/FOXP3+ ratio						
≤0.1250	39	24 (62)	119.64 (92.81–146.48)	0.47	1.24	0.68–2.61
>0. 1250	86	48 (56)	128.83 (105.59–152.07)			
Stromal CD20+/CD68+ ratio						
≤0.0997	63	37 (59)	128.03 (100.77–155.29)	0.80	0.93	0.54–1.60
>0.0997	62	35 (57)	134.15 (107.83–160.46)			
Tumoral CD20+/CD68+ ratio						
≤0.04	62	32 (52)	103.62 (81.83–125.42)	0.14	0.66	0.38–1.15
>0.004	63	40 (64)	145.68 (118.96–172.37)			
Stromal CD20+/CD163+ ratio						
≤0.0781	63	37 (59)	128.43 (101.31–155.54)	0.86	0.95	0.55–1.63
>0.0781	62	35 (57)	133.70 (107.22–160.18)			
Tumoral CD20+/CD163+ ratio						
≤0.0061	61	33 (54)	107.44 (85.25–129.63)	0.31	0.76	0.44–1.30
> 0. 0061	64	39 (61)	140.47 (114.06–166.88)			

Table S4. Stratified univariate Kaplan-Meier analysis to assess significant relationships of tumoral CD20⁺, clinicopathological and molecular variables on DSS in 125 OSCC patients.

Parameter	CD20⁺ Median	Number	Censored patients (%)	DSS (95% CI)	*P*
Age					
<65 years	≤0.33	38	18 (47)	102.02 (75.38–128.67)	0.03
	>0.33	39	28 (72)	168.57 (138.01–199.13)	
≥65 years	≤0.33	19	10 (53)	71.17 (42.86–99.49)	0.24
	>0.33	29	16 (55)	99.21 (74.51–123.92)	
Gender					
Female	≤0.33	17	10 (59)	116.40 (75.59–157.20)	0.87
	>0.33	26	15 (58)	127.37 (87.06–167.77)	
Male	≤ 0.33	40	18 (45)	91.40 (65.78–117.01)	0.02
	> 0.33	42	29 (69)	158.66 (127.24–190.07)	
Tobacco					
No	≤0.33	14	10 (71)	119.58 (78.32–160.84)	0.47
	>0.33	27	14 (52)	100.17 (72.24–128.10)	
Yes	≤0.33	43	18 (42)	90.44 (65.28–115.60)	0.004
	>0.33	41	30 (73)	164.76 (132.83–196.70)	
Alcohol					
No	≤0.33	20	12 (60)	102.94 (67.33–138.55)	0.97
	>0.33	36	19 (53)	123.92 (90.80–157.05)	
Yes	≤0.33	37	16 (43)	93.48 (66.44–120.52)	0.005
	>0.33	32	25 (78)	174.84 (140.22–209.47)	
pT					
T 1 + T2	≤0.33	35	21 (60)	118.94 (90.87–147.00)	0.20
	>0.33	46	32 (70)	163.15 (134.90–191.39)	
T 3 + T4	≤0.33	22	7 (32)	62.85 (32.71–92.99)	0.20
	>0.33	22	12 (55)	85.20 (55.70–114.70)	
pN					
N0	≤0.33	33	19 (58)	116.72 (88.11–145.33)	0.30
	>0.33	43	30 (70)	126.67 (106.30–147.03)	
N+	≤0.33	24	9 (38)	65.81 (35.35–96.27)	0.09
	>0.33	25	14 (56)	132.89 (90.81–174.98)	
Stage					
I + II	≤0.33	22	14 (64)	132.13 (100.24–164.02)	0.20
	>0.33	30	22 (73)	135.78 (114.71–156.85)	

III + IV	≤0.33	35	14 (40)	71.27 (45.58–96.96)	0.07
	>0.33	38	22 (58)	134.86 (99.96–169.76)	
Grade					
Well	≤0.33	33	15 (45)	84.41 (61.60–117.23)	0.08
	>0.33	47	29 (62)	143.87 (113.17–174.58)	
Moderate-poorly	≤0.33	24	13 (54)	109.75 (74.87–144.63)	0.22
	>0.33	21	15 (71)	125.89 (95.76–156.02)	
Site					
Tongue	≤0.33	23	11 (48)	90.59 (55.98–125.20)	0.33
	>0.33	28	17 (61)	135.99 (94.53–177.44)	
Other	≤0.33	34	17 (50)	104.08 (75.33–132.83)	0.09
	>0.33	40	27 (68)	126.23 (105.59–146.86)	
Radiotherapy					
No	≤0.33	21	17 (81)	155.63 (124.71–186.56)	0.22
	>0.33	29	27 (93)	159.74 (142.75–176.73)	
Yes	≤0.33	36	11 (31)	66.48 (43.57–89.39)	0.12
	>0.33	39	17 (44)	112.69 (80.85–144.53)	
PD-L1					
≤10%	≤0.33	49	27 (55)	110.66 (86.33–134.99)	0.24
	>0.33	55	36 (66)	150.75 (122.85–178.64)	
>10%	≤0.33	7	1 (14)	16.17 (6.59–25.74)	0.008
	>0.33	11	6 (55)	94.81 (54.41–135.22)	
SOX2					
Negative	≤0.33	25	9 (36)	70.02 (38.21–101.83)	0.01
	>0.33	47	28 (60)	134.65 (103.10–166.20)	
Positive	≤0.33	31	19 (61)	121.59 (92.03–151.15)	0.32
	>0.33	18	13 (72)	167.93 (127.86–208.01)	
NANOG					
Negative	≤0.33	35	15 (43)	85.89 (57.28–114.50)	0.03
	>0.33	48	30 (63)	143.79 (113.07–174.52)	
Positive	≤0.33	20	11 (55)	97.68 (68.72–126.65)	0.39
	>0.33	19	13 (68)	126.95 (94.90–159.00)	
Tumoral $CD8^+$ TIL					
≤24.65	≤0.33	36	19 (53)	104.44 (76.43–132.46)	0.33
	>0.33	25	16 (64)	149.32 (108.38–190.26)	
>24.65	≤0.33	21	9 (43)	83.16 (46.99–119.33)	0.03
	>0.33	43	28 (65)	114.07 (94.33–133.80)	

Stromal CD8⁺ TIL					
≤118	≤0.33	44	21 (48)	93.62 (67.85–119.40)	0.46
	>0.33	19	11 (58)	129.19 (81.85–176.53)	
>118	≤0.33	13	7 (54)	109.14 (64.89–153.40)	0.32
	>0.33	49	33 (67)	156.70 (127.92–185.48)	
Tumoral CD4⁺ TIL					
≤2.666	≤0.33	35	16 (46)	94.18 (67.62–120.75)	0.14
	>0.33	24	15 (63)	143.41 (104.49–182.34)	
>2.666	≤0.33	22	12 (55)	103.19 (65.05–141.33)	0.23
	>0.33	44	29 (66)	150.28 (118.18–182.38)	
Stromal CD4⁺ TIL					
≤32.66	≤0.33	39	18 (46)	96.37 (69.14–123.60)	0.03
	>0.33	23	16 (70)	159.58 (121.89–197.27)	
>32.66	≤0.33	18	10 (56)	111.68 (74.31–149.04)	0.64
	>0.33	45	28 (62)	141.54 (109.47–173.62)	
Tumoral FOXP3⁺ Tregs					
≤0.8333	≤0.33	31	13 (42)	83.25 (54.17–112.33)	0.16
	>0.33	31	17 (55)	123.72 (87.69–159.76)	
>0.8333	≤0.33	26	15 (58)	118.77 (86.42–151.13)	0.23
	>0.33	37	27 (73)	168.34 (136.25–200.44)	
Stromal FOXP3⁺ Tregs					
≤5.6667	≤0.33	35	14 (40)	80.82 (53.99–107.65)	0.06
	>0.33	28	17 (61)	116.20 (88.52–143.89)	
>5.6667	≤0.33	22	14 (64)	128.84 (94.45–163.22)	0.70
	>0.33	40	27 (68)	153.58 (120.65–186.51)	
Tumoral CD68⁺ macrophages					
≤36.1667	≤0.33	34	13 (38)	83.23 (56.17–110.30)	0.03
	>0.33	29	18 (62)	147.28 (110.31–184.26)	
>36.1667	≤0.33	23	15 (65)	121.95 (87.65–156.24)	0.74
	>0.33	39	26 (67)	114.70 (93.39–136.00)	
Stromal CD68⁺ macrophages					
≤108.000	≤0.33	40	19 (48)	92.74 (65.72–119.77)	0.24
	>0.33	16	10 (63)	140.87 (91.37–190.37)	
>108.000	≤ 0.33	17	9 (53)	108.18 (69.83–146.54)	0.36
	>0.33	52	34 (65)	151.42 (122.78–180.07)	
Tumoral CD163⁺ macrophages					
≤25.5833	≤0.33	37	19 (51)	105.70 (78.31–133.08)	0.25

	>0.33	27	18 (67)	152.69 (112.65–192.74)	
>25.5833	≤0.33	20	9 (45)	79.15 (43.94–114.36)	0.06
	>0.33	41	26 (63)	111.93 (91.72–132.15)	
Stromal CD163⁺ macrophages					
≤147.3333	≤0.33	37	18 (49)	101.02 (73.08–128.95)	0.25
	>0.33	24	15 (63)	111.00 (83.98–138.02)	
>147.3333	≤0.33	20	10 (50)	99.84 (63.29–136.38)	0.15
	>0.33	44	29 (66)	149.72 (117.98–181.46)	
